# Response of Flavor Substances in Tomato Fruit to Light Spectrum and Daily Light Integral

**DOI:** 10.3390/plants12152832

**Published:** 2023-07-31

**Authors:** Xiaoxue Fan, Na Lu, Wenshuo Xu, Yunfei Zhuang, Jing Jin, Xiaojuan Mao, Ni Ren

**Affiliations:** 1 Institute of Agricultural Information, Key Laboratory of Intelligent Agricultural Technology (Changjiang Delta), Ministry of Agriculture and Rural Affairs, Jiangsu Academy of Agricultural Sciences, Nanjing 210014, China; fxx@jaas.ac.cn (X.F.); jinjing@jaas.ac.cn (J.J.); 20190033@jaas.ac.cn (X.M.); 2 Center for Environment, Health and Field Sciences, Chiba University, 6-2-1 Kashiwanoha, Kashiwa 277-0882, Chiba, Japan; xuwenshuo1988@163.com (W.X.); zhuangyunfei1015@outlook.com (Y.Z.)

**Keywords:** PFALs, light quality, DLI, fruit quality, flavor substances

## Abstract

Light-emitting diodes (LEDs) have been widely used as light sources for plant production in plant factories with artificial lighting (PFALs), and light spectrum and light amount have great impacts on plant growth and development. With the expansion of the product list of PFALs, tomato production in PFALs has received attention, but studies on fruit quality influenced by artificial light are lacking. In this study, precisely modulated LED light sources based on white light combined with additional red, blue, and green lights were used to investigate the effects of light spectrum and daily light integral (DLI) on the main quality indicators and flavor substances of “Micro-Tom” tomato fruits. The highest sugar–acid ratio was obtained under the white light with addition of red light with high DLI and blue light with low DLI. The contents of β-carotene, lycopene, and lutein were significantly increased by higher DLI conditions except for under the blue light treatment, and the cross-interactions between the light spectrum and DLI were observed. The accumulation of the main flavor substances in tomato fruits was decreased by addition of green light with a high DLI and red light with a low DLI; notably, the percentage of 2-isobutylthiazole, which is associated with fresh tomato aroma, was decreased by green light. This study provides insights for improving tomato fruit quality and flavor by regulating light conditions in PFALs.

## 1. Introduction

Tomato (*Lycopersicon esculentum* Mill.) is a worldwide popular vegetable and is rich in nutrients such as vitamins, minerals, phenolic content, flavonoids, dietary fiber, and protein. It has been reported that the high amounts of lycopene, citric acid, and malic acid in tomato fruits have anticancer properties [1,2] and also can reduce the risk of hypertension and other cardiovascular diseases [3]. According to the Food and Agriculture Organization of the United Nations (FAOSTAT), the amount of cropland used for tomato production was 5.17 M ha, and tomato production reached 189.13 Mt in 2021 [4], which has an important position in the world vegetable production and supply. In recent years, tomato production inside plant factories with artificial lighting (PFAL) has been attracting more and more attention [5].

Light is an essential environmental factor affecting plant morphogenesis and physiological metabolism, which regulates plant growth and development due to three main aspects: light quality, light intensity, and photoperiod [6]. Daily light integral (DLI) is the product of the combination of light intensity and photoperiod and can be used as a light variable for plant growth instead of light intensity or photoperiod alone [7,8]. The four light aspects can be precisely controlled inside plant factories for crop production [9,10]. Light quality affects secondary metabolism, which is indispensable for plant life and closely connected to plant growth and environmental factors [11,12]. Most of the substances affecting fruit flavor belong to secondary metabolites, and their synthesis are sensitive to light quality [13,14]. It was reported that red light significantly increased the chestnut-like aroma and reduced the “raw grass” odor in green tea and the content of ketones, which may be responsible for the chestnut flavor of green tea [15]. In strawberry, the production of volatiles was influenced by light intensity throughout fruit development [16], and red light, blue light, and possibly far-red light are involved in regulating the synthesis of ethylcaproate, ethyl butyrate, and other ester substances [17].

In tomatoes, it has been found that the use of monochromatic red light, blue light, and combined red + blue light significantly improved tomato fruit quality indicators [18,19,20]. Yang et al. reported that combined red + blue light (R:B = 2:1, R:B = 1:1) significantly increased ascorbic acid, soluble protein content, soluble sugar mass fraction content, and lycopene content, and monochromatic red light significantly increased the soluble solids content of tomato fruits [18]. Li et al. reported that monochromatic red light and red + blue combination light (R:B = 3:1) significantly increased soluble sugar mass fraction, sugar-acid ratio, and lycopene content of tomato fruits, and monochromatic blue light significantly increased ascorbic acid content [19].

In addition to sugar and acid, an abundance of volatile compounds makes the tomato’s unique flavor, but only a limited number of volatile substances affect the aroma of tomato fruit, such as hexanal, β-ionone, cyclocitral, etc. [11,21]. To enhance the accumulation and percentage of these kinds of volatile substances is very important for producing good taste and preferable flavor tomatoes. The flavor and texture of tomatoes can be regulated by different cultivation techniques. Supplemental CO_2_ combined with supplemental light can enhance the content of volatile aromatic substances such as 6-methyl-5-hepten-2-one, ionone, and hexanal, which make tomato fruity and provide its aroma-rich flavor [11]. Spraying nano selenium can alleviate the abiotic stress caused by penthiopyrad on tomato fruits, regulating the levels of characteristic flavor substances such as hexanal, 6-methyl-5-hepten-2-one, 2-isobutylthiazole, eugenol, and β-ionone, improving fruit yield and flavor [21]. A study also demonstrated that combined red + blue light (R:B = 3:1) promoted ethylene biosynthesis and signaling through the regulation of melatonin content, which increased fruit softening, respiration rates, and carbohydrate and lycopene accumulation; promoted tomato fruit ripening; and delayed fruit senescence through the phytochrome signaling pathway [20]. After red light treatment, 3-methyl-1-butanol and 2-methyl butanal, which contribute to sweet flavor perception, are increased, suggesting that altering the ripening light environment can lead to a significant change in the volatiles that contribute to the flavor of tomatoes [17]. 

The previous studies on the light spectrum mainly focused on monochromatic red light, monochromatic blue light, and different ratios of combined red + blue light because it has been noted that most of the blue and red light can be absorbed by plant at once, and red + blue light combinations play the most important role in the growth and development of tomato [18,19,20,21,22,23], whereas few studies have paid attention to white light and other spectra. Some studies have revealed that monochromatic light cannot meet the light requirements for optimal plant growth [24,25,26]. Due to the lack of other wavebands, monochromatic light or different ratios of red + blue combined light do not induce a response from some photoreceptors in plants, which may hinder plant growth and development [27,28,29,30,31]. Therefore, studies on the effects of monochromatic wavelengths with a white light base on tomato fruit yield and taste would be more practical for determining the optimal light source for tomato production in PFALs.

In this study, the effects of different light aspects on the fruit quality of indoor cultivated tomato were investigated by using white light with the addition of different wavelengths of red, blue, and green lights, combined with different daily light integrals. This study aimed to explore the optimal light conditions required to produce good-flavored tomatoes in PFALs.

## 2. Results

### 2.1. Effects of Light Spectrum and DLI on the Growth of Tomato Plants

As shown in Table 1, the nitrogen balance index (NBI) and chlorophyll index of tomato leaves were not significantly affected by the light treatments. The flavonoids were promoted by additional blue light, especially when the DLI was low. The proanthocyanidin index was reduced by additional red light when the DLI was high as compared to other light treatments. The light spectrum and DLI have a significant effect on the flavonoid index and proanthocyanidin index as well as on cross-interaction.

### 2.2. Effects of Light Spectrum and DLI on Tomato Fruit Quality

The soluble sugar content was the highest under B-LD and R-HD treatments, followed by the W-HD treatment, and was the lowest under G-HD treatment (Table 2). In addition to the white light treatment, the soluble sugar of tomato fruits under the addition of red, blue, and green light spectra had significant differences at two levels of DLI, which indicated that different DLI levels had significant effects on the soluble sugar of tomato fruits; however, the effects of DLI depended on the light spectrum. The titratable acid content was increased by addition of blue light regardless of DLI level and was the highest under G-HD treatment and the lowest under the W-LD condition. The R-LD also resulted in higher titratable acid in tomato fruits than in the tomatoes under white lights. Except for under the red light treatment, the sugar–acid ratio was higher under high-DLI than under the low-DLI level. Interestingly, the titratable acid and sugar–acid ratio showed opposite effects by the DLI level. The vitamin C contents were higher under the high-DLI than under the low-DLI level for blue light and red light groups; however, this trend was opposite for white light groups. The light spectrum and DLI have a significant effect on the contents of soluble sugar, titratable acid, and vitamin C as well as on cross-interaction.

**Figure 1:**
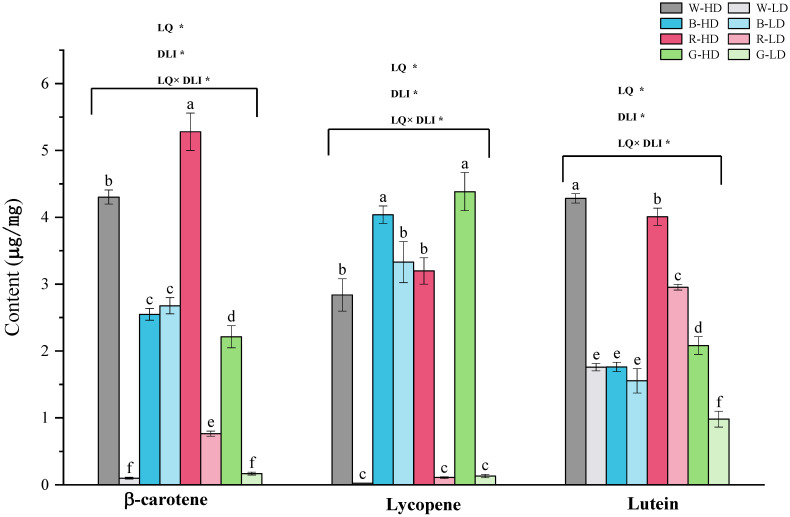
β-carotene, lycopene, and lutein contents under different light treatments. Different letters above the bar indicate significant differences (*p* < 0.05). The treatment marks, namely W-HD, W-LD, B-HD, B-LD, R-HD, R-LD, G-HD, and G-LD, represent the light spectra of white, blue, red, and green, with a high or a low daily light integral at 12.96 or 6.48 mol m^−2^ d^−1^, respectively. The * indicates significant differences (*p* < 0.05). LQ, light quality; DLI, daily light integral.

Figure 1 shows that β-carotene, lycopene, and lutein contents were significantly influenced by different light treatments. There were also significant interactive effects between light quality and DLI on these three parameters. We found that, except for the blue light treatment, the contents of β-carotene, lycopene, and lutein were significantly higher at higher-DLI compared to the lower-DLI treatments. The highest β-carotene content was found at R-HD, and the lowest was under the W-LD and G-LD treatments. The highest lycopene content was under G-HD and B-HD, and the lowest was under W-LD, R-LD, and G-LD. The lutein content in tomato fruits was the highest under W-HD, followed by R-HD, and the lowest under G-LD. 

### 2.3. Effect of Light Spectrum and DLI on Flavor Substance Content of Tomato Fruits

In this experiment, a total of 46 volatile substances were identified in tomato fruits under different light treatments using the GC-MS instrument, including 18 aldehydes, 8 ketones, 8 esters, 5 alcohols, and 7 other types of substances. These volatile substances contributed differently to the flavor of tomatoes due to the different structures and physicochemical properties of the flavor substances (Table A1). The relative content of aldehydes was the highest in all groups of tomatoes, followed by ketones and esters, and the lowest was alcohols and other chemical compositions. The analysis of the various types of compounds yielded that those aldehydes accounted for the largest proportion of the overall volatile compounds, reaching 41.10–49.06% in the different light treatments, followed by ketones with 25.60–43.20%, the esters with 5.80–24.78%, and other chemical compositions including hexadecane, 2-isobutylthiazole, and so on, with 6.63–17.61%; alcohols were lower, below 5%, and contributed less to the fruit flavor. Except for 2-nonenal, 2,4-nonadienal, and 4-oxononanal, the percentage of the content of aldehydes did not differ significantly among the light treatments, with the highest content under the white light in the low-level DLI treatment.

**Figure 2:**
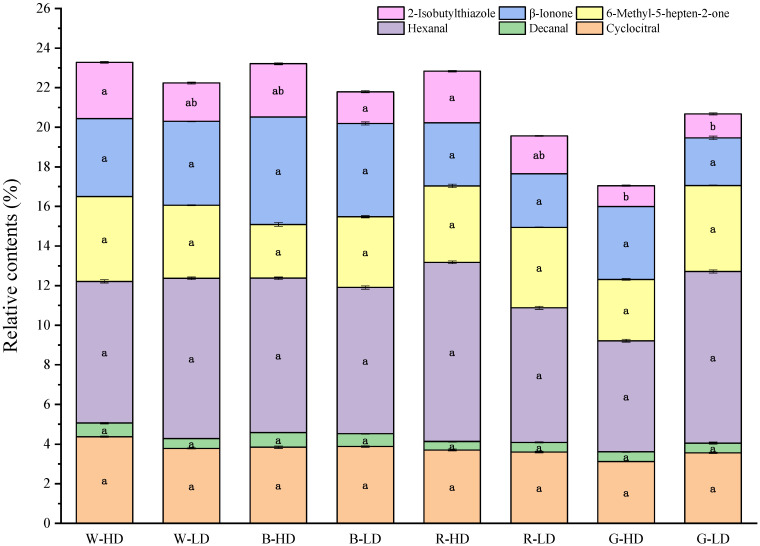
Relative contents of six typical flavor substances of tomato fruits under different light treatments. Different letters in the bar indicate significant differences (*p* < 0.05). The treatment marks, namely W-HD, W-LD, B-HD, B-LD, R-HD, R-LD, G-HD, and G-LD, represent the light spectra of white, blue, red, and green, with a high or a low daily light integral at 12.96 or 6.48 mol m^−2^ d^−1^, respectively.

Furthermore, Figure 2 shows the relative contents of six typical flavor substances that are considered the important substances that contribute to preferable aroma and flavor in tomato [32,33,34,35,36]. The accumulation of the six substances did not exceed 25% in all treatments, with hexanal accounting for the highest percentage. Among them, β-ionone, 6-methyl-5-hepten-2-one, hexanal, decanal, and cyclocitral did not show significant differences under different light treatments. However, 2-isobutylthiazole exhibited significant differences under different light treatments, with the lowest content under the green light treatments. The mean of total amount of the six substances was the highest in W-HD (23.3%) and the lowest in G-HD (17.0%).

### 2.4. Comprehensive Evaluation

Different light spectra and DLIs had various effects on tomato fruit quality indexes, leaf pigment content, and fruit flavor indexes in this study. It is difficult to comprehensively evaluate the effects of these light aspects on tomato fruit quality according to only one certain index. In this study, the entropy method was used to determine the membership function values of eight main indicators, including tomato fruit quality index, pigment content, and secondary metabolites that are considered as positively affecting taste and flavor. The coefficient of variation method was used to determine the weight of each index, and finally, the comprehensive score of each light treatment was calculated. The scores were sorted to find the best light conditions for improving tomato fruit quality and flavor. As shown in Table 3, the top three light treatments that were most favorable to improve the quality and flavor of tomato fruits were R-HD, B-LD, and B-HD, respectively, and their comprehensive scores were 1.676, 1.031, and 0.889, respectively.

## 3. Discussion

### 3.1. Effect of Light Spectrum and DLI on Growth of Tomato Plants

The NBI is the ratio of chlorophyll to flavonoids, which is used to rapidly assess the nitrogen nutrient status of plant leaves. NBI is one of the important indicators of C/N allocation changes [37,38]. In this study, there was no significant difference in the NBI of tomato plants under different light treatments, and all the NBI fell in the range of 48~53, indicating that tomato leaves had a high capacity to assimilate nitrogen, and were conducive to its protein synthesis and primary metabolism. Therefore, tomato plants had good performance under the light treatments used in the present study. Moreover, a small amount of additional blue light (B-LD) promoted flavonoid content in leaves compared to the white light. Interestingly, different from the blue light, a high amount of additional red light tended to promote the flavonoid content, although the difference between R-HD and R-LD was not significant (Table 1). However, high amounts of red light (R-HD) significantly reduced the production of proanthocyanin in tomato leaves, probably due to the high red light having the effect of promoting leaf expansion [39].

### 3.2. Effect of Light Spectrum and DLI on Nutritional Quality and Flavor Index of Tomato

The sugar–acid ratio index and flavor substances in tomato fruits play an important role in the taste of tomatoes. The soluble sugar content affects tomato flavor texture and is also an important quality indicator [35,40]. Tomato fruit sweetness is related to fruit sugar content but is also influenced by the content of titratable acids [41]. Studies reported that red light and combined red + blue light treatments could significantly increase the sugar and acid content of tomato fruits, and the highest sugar–acid ratio was found under combined red + blue light [11,19]. In this study, we found that the soluble sugar content was increased at high levels of DLI in red light and at low levels of DLI in blue light (Table 2). It may relate to the fact that red and blue light can affect leaf carbohydrate synthesis by promoting the activity of the key enzymes of sugar metabolism [20,21]. Titratable acid ratios increased with the addition of red, blue, and green light. After calculating the sugar–acid ratio, the highest sugar–acid ratios were found under W-LD, W-HD, R-HD, and B-LD treatments, and the sweet taste of the tomatoes was more intense. The addition of R, B, and G could change the acidity of tomato fruits, to different degrees, compared with white-based light (Table 2). Therefore, light spectra can be used to adjust the taste of tomatoes to meet different preferences of consumers.

The vitamin C content is one of the important indicators for evaluating the nutritional quality of tomato fruits [42]. In this study, the vitamin C content in tomato fruits was higher under high DLIs, except for under the white light treatment (Table 2). Several experiments investigating the effect of LED light on tomato quality have found that monochromatic red light and combined red + blue light can increase the vitamin C content in tomato fruits [18,19]. However, there was no significant change in the vitamin C content in tomato fruits after adding red or blue light spectra in this study. 

The content of β-carotene, lycopene, and lutein contributes significantly to the nutritional value of tomato fruits. Specifically, lycopene, a type of carotenoid, has anticancer, antioxidant, and immune-enhancing properties and is one of the key indicators of the nutritional quality of tomatoes [43]. Some other volatile and nonvolatile compounds that are essential for tomato flavor are derived from a range of precursors, including carotenoids [44]. It was reported that red light can affect lycopene synthesis by regulating the activity of photosensitive pigments in the fruit, while the photosensitive pigment content affects ethylene synthesis, and ethylene content has an important effect on lycopene content [21]. In this study, we found that the β-carotene content was significantly increased by high-level red light based on white light (Figure 1). In recent years, the effects of green light as an auxiliary spectrum on plants have been widely reported [24,25,26], and most studies on green light focused on physiological mechanisms for plant growth and development, such as reversing the blue light effect, reducing stomatal opening, and increasing the electrical conductivity of leaf flesh cells, with less research on this aspect of green light on pigment synthesis. This study shows that the addition of green light and blue light significantly enhanced lycopene, which contributes to the nutritional quality of tomato fruits.

Tomato flavor is a complex mixture of aromatic substances with volatile properties within the fruit, which is one of the most important factors for which consumers are willing to pay [36]. Many studies on tomato volatiles have been conducted in recent years, and more than 400 volatiles having been detected in different tomato varieties, mainly including aldehydes, ketones, alcohols, hydrocarbons, and esters. However, less than 30 volatiles mainly affect the flavor of tomato fruits [45]. The highest proportion of aldehydes is trans-2-hexenal, followed by n-hexenal, two aldehydes that usually relate to “grassy and raw green flavors” in tomato fruits [46]. This study identified a total of 46 types of volatile compounds, among which the total content of aldehydes and ketones was. High and had important impacts on the tomato flavor; meanwhile, the content of 2-hexenal was the highest, which contributes to fragrance (Table A1). It was found that flavor substances such as hexanal, heptanal, decanal, and cis-2-heptenal are associated with aroma and sweetness in tomatoes [34,36]. As introduced previously, in our study, we detected the six preferable flavor compounds that significantly influence tomato aroma and taste, and the highest proportion was hexanal, which is a substance that gives a green apple aroma. Among them, 2-isobutylthiazole (which gives the fresh tomato aroma) was significantly reduced by the addition of green light treatment (Figure 2).

In addition to flavor and taste, the nutrition level of tomato fruit, such as its contents of vitamin C, β-carotene, lycopene, and lutein, provides essential nutrients for the human body [47] and is also an important indicator to measure the quality of tomatoes, thus influencing the consumer’s decision. In this study, the comprehensive evaluation, including flavor and nutrition of the effects of light spectrum and DLI on tomato fruit quality and flavor, is shown in Table 3. The R-HD gained the highest score, which made great contributions to soluble sugar, β-carotene, vitamin C content, and total preferable flavor substances, followed by B-LD and B-HD. Previous studies reported that the quality and flavor of tomato fruit were promoted by red or red and blue light [48,49,50,51], possibly because red and blue light are the two major types of light driving photosynthate biosynthesis, and they are proven to be effective in fortifying the levels of plant nutrients and bioactive compounds in vegetables [52,53,54].

## 4. Materials and Methods

### 4.1. Materials

The experiment was carried out in a plant factory with artificial lighting in the Jiangsu Academy of Agricultural Sciences, Nanjing China. Tomato seeds (Micro Tom, PanAmerican Seed., West Chicago, IL, USA) were sown into plug trays filled with substrates of peat:perlite:vermiculite (volume ratio 3:1:1). After germination, the seedlings were put under white LED light with a light intensity (photosynthetic photon flux density, PPFD) of 300 μmol m^−2^ s^−1^. Temperature, photoperiod, CO_2_ concentration, and relative humidity were set to 26 ± 2 °C/18 ± 2 °C (light/dark), 12/12 h (light/dark), 500 ± 50 μmol/mol, and 60–80%, respectively. The seedlings were transplanted into pots (18 cm × 14 cm) filled with substrates of peat:perlite:vermiculite (volume ratio 3:1:1). The plants were irrigated with nutrient solution by an automatic system for 20 min per day. The fertilizer formula specialized for tomatoes was used, and the composition is as follows: 5Ca(NO3)_2_·NH_4_NO_3_·10H_2_O 80.13%, KNO_3_ 52.24%, EDTA-Fe 1.91%, KH_2_PO_4_ 16.2%, K_2_SO_4_ 5.02%, MgSO_4_·7H_2_O 44%, ZnSO_4_·7H_2_O 0.096%, Na_2_B_8_O_13_·4H_2_O 0.29%, MnSO_4_ 0.1%, CuSO_4_ 0.01%, and Na_2_MoO_4_ 0.008% (Shanghai Yongtong Ecological Engineering Co., Ltd., Shanghai, China). The EC and pH of the nutrient solution for seedlings were adjusted to 1.5 mS cm^−1^ and 6.0; for the flowering period, they were adjusted to 2.0 mS cm^−1^ and 6.0; and for the fruiting stage, they were adjusted to 3.08 mS cm^−1^ and 5.06, respectively, and the irrigation time was 15 min per day. At 20 days after sowing, six uniform seedlings with three true leaves were subjected to different light treatments for another 80 days. Temperature, photoperiod, CO_2_ concentration, and relative humidity were set to 26 ± 2 °C/18 ± 2 °C (light/dark), 12/12 h (light/dark), 500 ± 50 μmol/mol, and 60–70%, respectively.

### 4.2. Light Treatments

**Figure 3:**
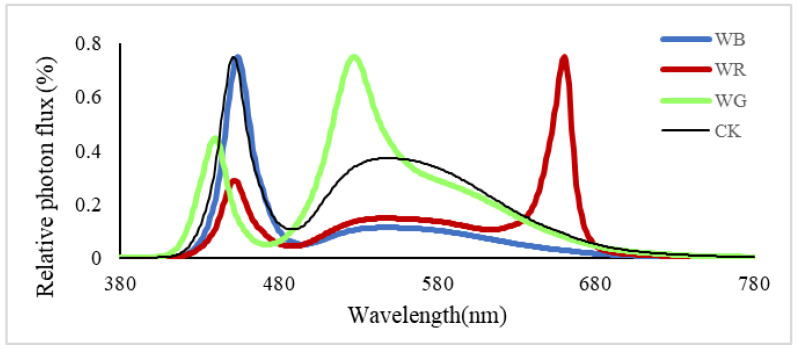
Relative spectral distribution of white (W/CK), white + blue (B), white + red (R), and white + green (G) lights used in this study.

The light sources are customized and precisely modulated with blue (B), green (G), red (R), and white (W) LED chips and were square-shaped with a dimension (length × width × height) of 680 mm × 330 mm × 180 mm (Nanjing Yunfang Agricultural Technology Co., Ltd., Nanjing, China). Each lamp used white LED chips as the basic light spectrum (B:G:R = 33:48:19) and added blue LED chips, green LED chips, or red LED chips to achieve the designed spectral requirements for the treatments. In total, three light spectrum treatments were designed: white + blue (B), white + red (R), and white + green (G). The white was set as the control (W/CK). The B, G, R ratio was 58:30:12 for B, 21:31:48 for R, 21:62:17 for G, and 33:48:19 for W, respectively. Two PPFD levels (150 ± 10 μmol m^−2^ s^−1^ and 300 ± 10 μmol m^−2^ s^−1^) were set for each spectrum condition to create two different DLI levels (see Table 4 for the light treatments settings). Two multispectral LED panels (max. 150 W each) were placed 20 cm above the crop level, and light intensity was adjusted to 300 μmol m^−2^ s^−1^. The spectral distributions of each treatment are shown in Figure 3. The photoperiod was 12 h per day.

### 4.3. Instruments and Measurement Methods

Three tomato plants were randomly selected from each experimental area for physiological index measurement, and the experiment was repeated three times. 

Three tomato plants were selected from each treatment to measure the biomass after harvesting. Shoot (stems and leaves) and root parts were separated, and the fresh mass of each part was measured with a 1/10,000 scale (Shimadzu, Japan). The dry mass of each part was measured after drying. The drying process was carried out using an oven (Shanghai Jinghong Experimental Equipment Co., Ltd., Shanghai, China). The material was first blanched at 110 °C and then dried at 90 °C for 15 h until it completely dried. Nitrogen utilization and pigment content indicators were measured non-destructively using a DUALEX Optical leafclip meter (Shanghai Zequan Technology Co., Ltd., Shanghai, China).

Measurement of fruit quality indicators: Six fruits were selected from each treatment. Three fruits with the same fruiting part were used for quality measurement in each treatment. Soluble protein was determined by the Coomassie brilliant blue G-250 method, soluble sugar was determined by the Anthrone colorimetric method, titratable acid was determined by the indicator titration method, and vitamin C was determined by the 2,6-dichlorophenol indophenol colorimetric method [6,55,56].

Flavor substances were detected by referring to the method of Han et al. [57]. Three fruits from each group of replications were ground, mixed, and stored in sample tubes for later use. Sample preparation: The dried tomato samples were crushed, and 2.0 g was placed in a 20 mL headspace vial, which was quickly sealed, and the headspace vial was placed in a thermostatic water bath. Then the aged solid-phase microextraction injection needle was inserted into a sealed headspace vial, and the extraction head was pushed out and extracted in a water bath at 50 °C for 1 h. After that, the extraction head was quickly removed and immediately inserted into the sample inlet of gas chromatograph–mass spectrometer (GC-MS) and thermally resolved for 2 min. The extraction head was aged at 250 °C for 5 min before each sample extraction to reduce the memory effect.

GC-MS analysis was under the following conditions: The column was a TG-5MS (30 m × 0.25 mm, 0.25 μm) elastic quartz capillary column. The carrier gas was high-purity helium (99.999%) with a flow rate of 1.2 mL/min and splitless mode. The sample inlet temperature was 250 °C. The GC temperature program started with an initial temperature at 40 °C held for 2 min and then raised to 100 °C (held for 1 min) at a rate of 3 °C/min, then raised to 160 °C (held for 1 min) at a rate of 5 °C/min, and finally raised to 280 °C (held for 1 min) at a rate of 10 °C/min. MS was under the following conditions: EI ion source; transfer line temperature, 280 °C; ion source temperature, 300 °C; ionizing energy, 70 eV. The full-scan range was *m*/*z* 33–800. Data acquisitions were carried out in the full-scan model.

High-performance liquid chromatography (HPLC) was used for the determination of β-carotene, lycopene, and lutein [58]. Carotenoids were extracted by acetone and were analyzed using Waters ACQUITY UPLC system. Fresh tomato material was frozen in liquid nitrogen and ground to a fine powder, and 2 g was weighed. Next, the sample was transferred to a test tube. For each tube, 10 mL of acetone was added and then was kept in an ultrasonic water bath for 15 min. Then, the solution was centrifuged with a high-speed centrifuge, and the supernatant was collected. The extraction was repeated until the samples became colorless. The supernatants from each extraction were collected together and taken to dryness on a rotary evaporator at 35 °C. The solid residue was re-dissolved in 1 mL of a methanol solution, and the solution was filtered through a 0.22 µm filter. This solution was then used for HPLC analysis.

LC column conditions were as follows: Waters Symmetry Shield RP18 reversed-phase column (4.6 mm × 250 mm, 5 μm); the column temperature was maintained at 30 °C. A mixed methanol/acetonitrile/dichloromethane (20/75/5, *v*/*v*/*v*) solution was used. The injection volume was 10 μL.

### 4.4. Calculation of Data

Qualitative analysis of flavor components: Through computer search and comparison with standard mass spectra provided by NIST 105 and Wiley 7.0 mass spectral libraries, the volatile components of tomato identified by GCMS analysis were analyzed to determine their chemical components.

Quantitative analysis of flavor components: The NIST spectral library workstation data processing system was used for quantitative analysis according to the peak area normalization method, and the percentage content of each chemical component in the volatile components of tomato was obtained.

The calculation method of daily light integral (DLI):

DLI = light intensity × photoperiod × 3600 × 10^−6^

where DLI is the daily cumulative light amount, mol/m^2^/d; the unit of light intensity is μmol/m^2^/s; and the unit of photoperiod is h/d [8].

Light intensity and spectrum measurement: light intensity was measured at 20 cm directly below the LED lamps using a portable light quantum meter (LI-1400, LI-COR, Lincoln, NE, USA), and the spectral distributions of light were measured by a spectrometer (AvaSpec-ULS2048, Avantes, NS Apeldoorn, The Netherlands) (Figure 3). According to the spectral distribution, the ratios of blue light (B, wavelength 400–499 nm), green light (G, wavelength 500–599 nm), and red light (R, wavelength 600–699 nm) were calculated, respectively.

### 4.5. Data Analysis

The processing analysis and chart drawing of the experimental data were completed using Microsoft Excel 2020 and SPSS 22.0 software. The variance analysis was based on the least significant difference method, and multiple comparisons were performed at the significance level of 0.05. The comprehensive evaluation of fruit flavor was conducted using the entropy method. The calculation method is as follows [59]:

(1) Index dimensionless processing:

(1) $$x\prime_{ij}=\frac{x_{ij}-m_{j}}{M_{j}-m_{j}}$$

where *M_j_* is the maximum value of *x_ij_*, and *m_j_* is the minimum value of *x_ij_*.

(2) Data standardization:

(2) $$p_{ij=\frac{x\prime_{ij}}{\sum_{i=1}^{n}x_{ij}}}$$

(3) Entropy value calculation of the *j_th_*:

(3) $$e_{i}=\frac{1}{\ln n}\sum_{i=1}^{n}p_{ij\;}\ln\left(p_{ij}\right),\;0\;\leq \;e_{i}\;\leq \;1$$

(4) Calculation of coefficient of difference:

*g_i_* = 1 − *e_i_*

(5) Determine the weight *w_j_* of the indicator *j* and calculate the comprehensive score:

(4) $$W_{j}=\frac{g_{i}}{\sum_{i=1}^{m}g_{i}},\;j=1,\;2,\;3\ldots m\;$$

## 5. Conclusions

This study demonstrates that light spectrum and DLI have significant interactive effects on tomato quality indicators, including soluble sugar, titratable acids, vitamin C, β-carotene, lycopene, and lutein contents in tomato fruits. The comprehensive evaluation reveals that the use of white light with the addition of red or blue light has a better promotional effect on the quality and taste of tomato fruit than green light, whereas results also depend on the DLI level. The addition of red light with a higher DLI gave the highest overall score, which significantly improved the sugar–acid ratio of tomato fruits and increased the pleasant taste and aroma. The addition of blue light with lower and higher DLIs achieved the second and the third overall scores, respectively. The addition of green light increased the sour taste and decreased positive flavor substances in tomato fruits. The results showed that tomato quality and flavor can be improved by adjusting light conditions with a suitable light spectrum and DLI level in a plant factory. However, the effects of light aspects on flavor substances in tomato fruit are rather complex, and further studies need to be conducted for a better understanding.

## Figures and Tables

**Table 1:** Effect of light spectrum and DLI on nitrogen utilization and pigment content of tomato leaves. Means ± SD. Different letters in the same column indicate significant differences (*p* < 0.05).

Treatment	Nitrogen Balance Index		Chlorophyll Index		Flavonoid Index		Proanthocyanidin Index	
W-HD	49.26 ± 3.14	a	40.73 ± 8.15	a	0.82 ± 0.12	b	0.12 ± 0.47	a
W-LD	51.01 ± 5.37	a	40.50 ± 2.75	a	0.80 ± 0.06	b	0.14 ± 0.03	a
B-HD	48.56 ± 1.08	a	48.24 ± 6.23	a	1.01 ± 0.16	ab	0.10 ± 0.05	ab
B-LD	45.80 ± 6.28	a	45.61 ± 8.36	a	1.12 ± 0.38	a	0.10 ± 0.06	ab
R-HD	48.16 ± 1.30	a	43.30 ± 7.48	a	0.92 ± 0.11	ab	0.07 ± 0.03	b
R-LD	49.03 ± 9.70	a	38.05 ± 5.90	a	0.79 ± 0.09	b	0.15 ± 0.04	a
G-HD	53.13 ± 7.51	a	41.23 ± 8.70	a	0.77 ± 0.07	b	0.11 ± 0.05	ab
G-LD	47.90 ± 7.57	a	37.93 ± 4.96	a	0.77 ± 0.05	b	0.15 ± 0.06	a
Light quality (LQ)	ns		ns		*		*	
Daily light integral (DLI)	*		*		*		*	
DLI×LQ	ns		ns		*		*	

Note: * indicates significant differences (*p* < 0.05); ns indicates no significant differences. The treatment marks, namely W-HD, W-LD, B-HD, B-LD, R-HD, R-LD, G-HD, and G-LD, represent the light spectra of white, blue, red, and green, with a high or a low daily light integral at 12.96 or 6.48 mol m^−2^ d^−1^, respectively.

**Table 2:** Effect of light spectrum and DLI on soluble sugar, titratable acid, sugar/acid ratio, and vitamin C of tomato fruit. Means ± SD. Different letters in the same column indicate significant differences (*p* < 0.05).

Treatment	Soluble Sugar (%)		Titratable Acid (%)		Sugar/Acid Ratio		Vitamin C (mg/100 g)	
W-HD	1.90 ± 0.09	ab	0.78 ± 0.03	de	2.43 ± 0.16	ab	22.21 ± 0.47	bc
W-LD	1.74 ± 0.20	abc	0.64 ± 0.04	e	2.73 ± 0.46	a	28.27 ± 0.90	a
B-HD	1.32 ± 0.28	bcd	1.23 ± 0.07	b	1.07 ± 0.19	cd	23.62 ± 4.30	ab
B-LD	2.06 ± 0.21	a	1.03 ± 0.04	bc	1.99 ± 0.13	ab	17.18 ± 4.05	cd
R-HD	2.05 ± 0.70	a	0.93 ± 0.13	cd	2.30 ± 0.06	ab	27.92 ± 2.79	a
R-LD	1.21 ± 0.42	cd	1.17 ± 0.03	b	1.04 ± 0.37	cd	16.39 ± 3.55	d
G-HD	0.74 ± 0.08	d	1.48 ± 0.29	a	0.52 ± 0.14	d	20.63 ± 1.27	bcd
G-LD	1.51 ± 0.06	abc	0.84 ± 0.05	cde	1.81 ± 0.16	bc	17.58 ± 0.50	cd
Light quality (LQ)	*		*		*		*	
Daily light integral (DLI)	*		*		ns		*	
DLI×LQ	*		*		*		*	

Note: * indicates significant differences (*p* < 0.05); ns indicates no significant differences. The treatment marks, namely W-HD, W-LD, B-HD, B-LD, R-HD, R-LD, G-HD, and G-LD, represent the light spectra of white, blue, red, and green, with a high or a low daily light integral at 12.96 or 6.48 mol m^−2^ d^−1^, respectively.

**Table 3:** Comprehensive evaluation for the effects of light spectrum and DLI on tomato fruit quality and flavor by entropy method. (The calculation details of this method are described in Section 4.5 of Section 4.)

Indicators	W-HD	W-LD	B-HD	B-LD	R-HD	R-LD	G-HD	G-LD
Soluble sugar content	0.033	0.067	0.07	0.078	0.259	0.094	0	0.018
Titratable acid	0.009	0	0.06	0.031	0.079	0.033	0.311	0.021
Sugar-to-acid ratio	0.033	0.101	0.027	0.026	0.223	0.051	0	0.032
Vitamin C content	0.012	0.036	0.127	0.018	0.111	0	0.025	0.003
β-carotene	0.146	0.125	0.08	0.127	0.159	0.004	0	0.104
Lycopene	0.002	0	0.255	0.088	0.047	0.002	0.007	0.044
Lutein	0.004	0.221	0.045	0.15	0.142	0.002	0	0.095
Total preferable flavor substances	0.418	0.321	0.226	0.512	0.655	0.366	0.265	0.11
**Comprehensive** **evaluation value**	0.658	0.871	**0.889**	**1.031**	**1.676**	0.553	0.608	0.427
**Order**	5	4	**3**	**2**	**1**	7	6	8

Note: The treatment marks, namely W-HD, W-LD, B-HD, B-LD, R-HD, R-LD, G-HD, and G-LD, represent the light spectra of white, blue, red, and green, with a high or a low daily light integral at 12.96 or 6.48 mol m^−2^ d^−1^, respectively. The numbers in bold are the rankings of the top three in the comprehensive evaluation.

**Table 4:** Light environmental parameters for tomato growth.

Light Treatment	Daily Light Integral	Main Peak Wavelength	Light Intensity	Photoperiod
	(mol m^−2^ d^−1^)	of the Spectrum (nm)	(μmol m^−2^ s^−1^)	(h d^−1^)
W-HD	6.48	380–780	150	12
W-LD	12.96		300	
B-HD	6.48	450–470	150	12
B-LD	12.96		300	
R-HD	6.48	620–640	150	12
R-LD	12.96		300	
G-HD	6.48	520–530	150	12
G-LD	12.96		300	

Note: The treatment marks, namely W-HD, W-LD, B-HD, B-LD, R-HD, R-LD, G-HD, and G-LD, represent the light spectra of white, blue, red, and green, with a high or a low daily light integral at 12.96 or 6.48 mol m^−2^ d^−1^, respectively.

## Data Availability

Not applicable.

## Appendix A

**Table A1:** Effect of light quality and DLI on the relative content of characteristic volatiles in tomato fruits. Means ± SD. Different letters in the same row indicate significant differences (*p* < 0.05).

Reservation Time (min)	Volatile Substance Composition	Chemical Formula	W-HD	W-LD		B-HD		B-LD		R-HD		R-LD		G-HD		G-LD		
100%																		
	**Aldehydes**																	
4.3	Hexanal	C_6_H_12_O	7.16 ± 0.01	a	8.10 ± 0.02	a	7.80 ± 0.01	a	7.39 ± 0.01	a	9.05 ± 0.01	a	6.80 ± 0.31	a	5.61 ± 0.02	a	8.66 ± 0.03	a
5.36	2-Hexenal	C_6_H_10_O	22.28 ± 0.11	a	29.45 ± 0.07	a	22.76 ± 0.07	a	20.49 ± 0.02	a	25.04 ± 0.02	a	26.24 ± 0.03	a	24.48 ± 0.08	a	21.01 ± 0.08	a
7.69	2-Heptenal	C_7_H_12_O	0.71 ± 0.01	a	0.39 ± 0.01	a	0.96 ± 0.01	a	0.66 ± 0.01	a	0.56 ± 0.01	a	0.53 ± 0.01	a	0.40 ± 0.01	a	0.65 ± 0.01	a
9.08	2,4-Heptadienal	C_7_H_10_O	0.41 ± 0.01	a	0.23 ± 0.02	a	0.45 ± 0.01	a	0.49 ± 0.05	a	0.30 ± 0.01	a	0.43 ± 0.01	a	0.35 ± 0.02	a	0.25 ± 0.01	a
9.51	2-Nonenal	C_9_H_16_O	0.07 ± 0.01	b	0.06 ± 0.01	b	0.22 ± 0.01	ab	0.27 ± 0.01	a	0.21 ± 0.01	a	0.05 ± 0.01	b	0.18 ± 0.01	ab	0.07 ± 0.01	b
10.12	5-Octen-2-yn-4-ol	C_8_H_12_O	0.21 ± 0.01	a	0.24 ± 0.01	a	0.17 ± 0.01	a	0.16 ± 0.01	a	0.16 ± 0.01	a	0.16 ± 0.01	a	0.17 ± 0.01	a	0.20 ± 0.01	a
10.22	2-Octenal	C_8_H_14_O	2.53 ± 0.02	a	1.33 ± 0.01	a	1.93 ± 0.01	a	1.67 ± 0.01	a	2.01 ± 0.01	a	2.27 ± 0.01	a	1.38 ± 0.01	a	2.3 ± 0.01	a
11.37	Nonanal	C_9_H_18_O	2.20 ± 0.01	a	1.31 ± 0.01	a	1.49 ± 0.01	a	1.20 ± 0.01	a	1.32 ± 0.01	a	1.21 ± 0.01	a	1.51 ± 0.01	a	1.79 ± 0.01	a
13.83	Decanal	C_10_H_20_O	1.27 ± 0.01	a	1.01 ± 0.01	a	1.17 ± 0.01	a	0.93 ± 0.01	a	0.70 ± 0.01	a	0.66 ± 0.01	a	1.21 ± 0.01	a	1.11 ± 0.01	a
14.06	2,4-Nonadienal	C_9_H_14_O	0.32 ± 0.01	ab	0.19 ± 0.01	ab	0.47 ± 0.01	ab	0.51 ± 0.01	a	0.17 ± 0.01	b	0.18 ± 0.01	b	0.30 ± 0.01	ab	0.21 ± 0.01	ab
14.26	1-Cyclohexene-1-carboxaldehyde	C_10_H_16_O	0.58 ± 0.01	a	0.59 ± 0.01	a	0.52 ± 0.01	a	0.51 ± 0.01	a	0.52 ± 0.01	a	0.38 ± 0.01	a	0.50 ± 0.01	a	0.46 ± 0.01	a
14.71	2,6-Octadienal	C_10_H_16_O	1.21 ± 0.01	a	1.14 ± 0.01	a	0.99 ± 0.01	a	1.20 ± 0.01	a	1.14 ± 0.01	a	1.11 ± 0.01	a	0.88 ± 0.01	a	1.01 ± 0.01	a
14.82	4-Oxononanal	C_9_H_16_O_2_	0.20 ± 0.01	ab	0.19 ± 0.01	ab	0.31 ± 0.01	a	0.26 ± 0.01	ab	0.16 ± 0.01	ab	0.16 ± 0.01	ab	0.20 ± 0.01	ab	0.14 ± 0.01	ab
15.16	2-Decenal	C_10_H_18_O	0.69 ± 0.01	a	0.48 ± 0.01	a	0.73 ± 0.01	a	0.64 ± 0.01	a	0.42 ± 0.01	a	0.48 ± 0.01	a	0.49 ± 0.01	a	0.49 ± 0.01	a
15.4	Cyclocitral	C_10_H_16_O	4.36 ± 0.01	a	3.79 ± 0.01	a	3.85 ± 0.01	a	3.88 ± 0.01	a	3.71 ± 0.01	a	3.60 ± 0.01	a	3.12 ± 0.01	a	3.56 ± 0.01	a
16.43	2,4-Decadienal	C_10_H_16_O	0.97 ± 0.01	a	0.37 ± 0.01	a	0.65 ± 0.01	a	0.64 ± 0.01	a	0.67 ± 0.01	a	0.74 ± 0.01	a	0.34 ± 0.01	a	0.61 ± 0.01	a
17.81	Hexadecyl 3-methylbut-3-enyl ester	C_25_H_44_O_4_	0.87 ± 0.01	a	0.80 ± 0.01	a	1.09 ± 0.01	a	1.07 ± 0.01	a	0.75 ± 0.01	a	0.60 ± 0.01	a	0.78 ± 0.01	a	0.60 ± 0.01	a
18.42	Dodecanal	C_12_H_24_O	0.22 ± 0.01	a	0.21 ± 0.01	a	0.22 ± 0.01	a	0.20 ± 0.01	a	0.18 ± 0.01	a	0.15 ± 0.01	a	0.21 ± 0.01	a	0.15 ± 0.01	a
19.64	8-Hexadecenal, 14-methyl-, (Z)-	C_17_H_32_O	0.28 ± 0.01	ab	0.19 ± 0.01	ab	0.37 ± 0.01	a	0.32 ± 0.01	ab	0.21 ± 0.01	ab	0.17 ± 0.01	ab	0.20 ± 0.01	ab	0.14 ± 0.01	b
	**Total**		**45.27**	**49.06**		**44.98**		**41.56**		**41.10**		**42.30**		**46.58**		**45.26**		
	**Ketone**																	
8.47	5-Hepten-2-one, 6-methyl-	C_8_H_14_O	4.29 ± 0.02	a	3.69 ± 0.02	a	2.72 ± 0.01	a	3.57 ± 0.01	a	3.86 ± 0.01	a	4.07 ± 0.01	a	3.09 ± 0.01	a	4.35 ± 0.02	a
12.42	8-Decen-2-one	C_12_H_20_O	0.19 ± 0.01	b	0.17 ± 0.01	b	0.18 ± 0.01	b	0.32 ± 0.01	b	0.20 ± 0.01	b	0.13 ± 0.01	b	0.14 ± 0.01	a	0.16 ± 0.01	b
18.89	α-Ionone	C_13_H_20_O	0.27 ± 0.01	a	0.16 ± 0.01	a	0.27 ± 0.01	a	0.24 ± 0.01	a	0.16 ± 0.01	a	0.12 ± 0.01	a	0.21 ± 0.01	a	0.09 ± 0.01	a
19.42	5,9-Undecadien-2-one, 6,10-dimethyl-	C_13_H_22_O	23.27 ± 0.04	ab	17.95 ± 0.02	b	21.4 ± 0.02	ab	23.47 ± 0.02	a	21.02 ± 0.02	ab	19.80 ± 0.02	ab	15.26 ± 0.09	b	14.59 ± 0.04	b
20.14	β-Ionone	C_13_H_20_O	3.94 ± 0.02	a	4.24 ± 0.02	a	5.43 ± 0.02	a	4.71 ± 0.01	a	3.20 ± 0.02	a	2.70 ± 0.02	a	3.67 ± 0.02	a	2.41 ± 0.01	a
21	3,5,9-Undecatrien-2-one, 6,10-dimethyl-, (E,Z)-	C_13_H_20_O	0.38 ± 0.01	ab	0.23 ± 0.001	ab	0.37 ± 0.01	ab	0.47 ± 0.01	a	0.28 ± 0.01	ab	0.26 ± 0.01	ab	0.23 ± 0.01	ab	0.18 ± 0.01	b
22.29	Propanoic acid	C_16_H_30_O_4_	0.33 ± 0.01	a	1.36 ± 0.02	a	0.43 ± 0.01	a	0.28 ± 0.01	a	0.32 ± 0.01	a	1.30 ± 0.02	a	1.05 ± 0.01	a	0.45 ± 0.01	a
27.99	5,9,13-Pentadecatrien-2-one	C_18_H_30_O	4.80 ± 0.02	ab	3.98 ± 0.03	ab	6.76 ± 0.01	ab	10.14 ± 0.01	a	7.01 ± 0.04	ab	4.37 ± 0.03	ab	5.66 ± 0.05	ab	3.37 ± 0.04	b
	**Total**		**37.47**	**31.78**		**37.56**		**43.2**		**29.31**		**25.6**		**36.05**		**32.75**		
	**Esters**																	
15.91	2,4-Dodecadienal	C_12_H_20_O	0.72 ± 0.01	a	0.23 ± 0.01	a	0.37 ± 0.01	a	0.38 ± 0.01	a	0.48 ± 0.01	a	0.60 ± 0.01	a	0.28 ± 0.01	a	0.53 ± 0.01	a
9.92	Phenylacetic acid	C_12_H_14_O_2_	0.18 ± 0.01	b	0.19 ± 0.01	b	0.31 ± 0.01	b	0.29 ± 0.01	b	0.30 ± 0.01	b	0.31 ± 0.01	b	0.27 ± 0.01	b	0.62 ± 0.01	a
7.84	E-2-Hexenyl benzoate	C_13_H_16_O_2_	1.11 ± 0.01	a	1.70 ± 0.01	a	1.13 ± 0.01	a	1.90 ± 0.01	a	1.67 ± 0.01	a	0.99 ± 0.01	a	1.05 ± 0.01	a	1.36 ± 0.01	a
11.12	Ethanone, 1-(1-cyclohexen-1-yl)	C_8_H_12_O	0.44 ± 0.01	ab	0.30 ± 0.01	ab	0.47 ± 0.01	b	0.63 ± 0.01	ab	0.70 ± 0.01	ab	0.44 ± 0.01	ab	1.03 ± 0.01	b	0.39 ± 0.01	a
12.73	6-Nonenal	C_9_H_16_O	1.44 ± 0.01	a	1.15 ± 0.01	a	0.91 ± 0.01	a	1.38 ± 0.01	a	0.76 ± 0.01	a	1.44 ± 0.01	a	1.62 ± 0.01	a	0.95 ± 0.01	a
12.88	10,13-Octadecadiynoic acid	C_19_H_30_O_2_	0.19 ± 0.01	b	0.25 ± 0.01	b	0.30 ± 0.01	ab	0.23 ± 0.01	b	0.32 ± 0.01	b	0.19 ± 0.01	b	0.46 ± 0.01	a	0.24 ± 0.01	b
13.66	Methyl salicylate	C_8_H_8_O_3_	5.48 ± 0.09	a	4.63 ± 0.07	a	3.04 ± 0.03	a	0.57 ± 0.01	a	5.78 ± 0.09	a	9.55 ± 0.10	a	18.65 ± 0.23	a	20.52 ± 0.13	a
16.18	Undecanal	C_11_H_22_O	0.31 ± 0.01	a	0.29 ± 0.01	a	0.37 ± 0.01	a	0.42 ± 0.01	a	0.32 ± 0.01	a	0.20 ± 0.01	a	0.27 ± 0.01	a	0.17 ± 0.01	a
	**Total**		9.87	8.74		6.90		5.80		23.63		24.78		10.33		13.72		
	**Alcohols**																	
11.3	1,6-Octadien-3-ol	C_10_H_18_O	0.34 ± 0.01	a	0.50 ± 0.01	a	0.20 ± 0.01	a	0.21 ± 0.01	a	0.31 ± 0.01	a	0.37 ± 0.01	a	0.35 ± 0.01	a	0.37 ± 0.01	a
17.71	cis-4,5-Epoxy-(E)-2-decenal	C_10_H_16_O_2_	0.30 ± 0.01	a	0.33 ± 0.01	a	0.42 ± 0.01	a	0.34 ± 0.01	a	0.27 ± 0.01	a	0.29 ± 0.01	a	0.32 ± 0.01	a	0.27 ± 0.01	a
20.9	trans-Geranylgeraniol	C_20_H_34_O	0.29 ± 0.01	ab	0.23 ± 0.01	ab	0.34 ± 0.01	ab	0.39 ± 0.01	a	0.27 ± 0.01	ab	0.20 ± 0.01	ab	0.22 ± 0.01	ab	0.15 ± 0.01	b
22.53	Cedrol	C_15_H_26_O	0.24 ± 0.01	a	2.39 ± 0.04	a	0.48 ± 0.01	a	0.25 ± 0.01	a	0.33 ± 0.01	a	1.89 ± 0.03	a	1.06 ± 0.02	a	0.43 ± 0.01	a
	**Total**		**2.44**	**4.46**		**2.61**		**2.12**		**3.16**		**2.33**		**1.88**		**3.42**		
	**Other chemical**																	
	**composition**																	
9.63	2-Isobutylthiazole	C_7_H_11_NS	2.84 ± 0.01	a	1.95 ± 0.01	ab	1.60 ± 0.01	ab	2.69 ± 0.02	a	2.60 ± 0.01	a	1.91 ± 0.01	ab	1.05 ± 0.01	b	1.20 ± 0.01	b
13.17	3-(1-Cyclopentenyl)furan	C_9_H_10_O	0.14 ± 0.01	a	0.10 ± 0.01	a	0.14 ± 0.01	a	0.15 ± 0.01	a	0.11 ± 0.01	a	0.08 ± 0.01	a	0.09 ± 0.01	a	0.10 ± 0.01	a
16.49	3-Nonen-5-yne, 4-ethyl-	C_11_H_18_	1.06 ± 0.01	ab	0.45 ± 0.01	b	1.58 ± 0.01	ab	1.93 ± 0.01	a	1.17 ± 0.01	ab	0.91 ± 0.01	ab	0.92 ± 0.01	ab	0.56 ± 0.01	b
17.45	3-(1-Cyclopentenyl)furan	C_11_H_20_O	0.66 ± 0.01	ab	2.28 ± 0.03	ab	3.19 ± 0.02	a	1.50 ± 0.01	ab	0.50 ± 0.01	b	1.18 ± 0.01	ab	0.66 ± 0.01	ab	0.38 ± 0.01	b
12.18	8-Decen-2-one, 9-methyl-5-methylene	C_10_H_18_	0.51 ± 0.01	c	0.54 ± 0.01	c	0.78 ± 0.01	ab	0.94 ± 0.01	a	0.59 ± 0.01	bc	0.62 ± 0.01	abc	0.56 ± 0.01	c	0.93 ± 0.01	ab
23.68	Hexadecane, 1,1-bis(dodecyloxy)-	C_40_H_82_O_2_	0.18 ± 0.01	ab	0.12 ± 0.01	ab	0.28 ± 0.01	a	0.26 ± 0.01	ab	0.18 ± 0.01	ab	0.10 ± 0.01	b	0.15 ± 0.01	ab	0.09 ± 0.01	b
25	2,6,10-Dodecatrienal, 3,7,11-trimethyl	C_15_H_24_O	4.80 ± 0.01	a	3.98 ± 0.01	a	6.76 ± 0.01	a	10.14 ± 0.01	a	7.01 ± 0.01	a	4.37 ± 0.01	a	5.66 ± 0.01	a	3.37 ± 0.01	a
	**Total**		**10.19**	**9.42**		**14.33**		**17.61**		**9.09**		**6.633**		**12.16**		**9.17**		

Note: The treatment marks, namely W-HD, W-LD, B-HD, B-LD, R-HD, R-LD, G-HD, and G-LD, represent the light spectra of white, blue, red, and green, with a high or a low daily light integral at 12.96 or 6.48 mol m^−^^2^ d^−^^1^, respectively. Names of volatile substance groups and totaled results are shown in bold.
